# The Widely Used Antihelmintic Drug Albendazole is a Potent Inducer of Loss of Heterozygosity

**DOI:** 10.3389/fphar.2021.596535

**Published:** 2021-02-18

**Authors:** Luiza S. E. P. Will Castro, Wietske Pieters, Mir Farshid Alemdehy, Muhammad A. Aslam, Olimpia Alessandra Buoninfante, Jonne A. Raaijmakers, Bas Pilzecker, Paul C. M. van den Berk, Hein te Riele, René H. Medema, Rozangela C. Pedrosa, Heinz Jacobs

**Affiliations:** ^1^ Division of Tumor Biology and Immunology, Netherlands Cancer Institute, Amsterdam, Netherlands; ^2^ Department of Biochemistry, Federal University of Santa Catarina, Florianópolis, Brazil; ^3^ Institute of Molecular Biology and Biotechnology, Bahauddin Zakariya University, Multan, Pakistan; ^4^ Division of Cell Biology, Netherlands Cancer Institute, Amsterdam, Netherlands

**Keywords:** albendazole, aneuploidy, helminth, spindle poison, loss of heterozygosity (LOH), cancer predisposition

## Abstract

The antihelmintic drug ABZ and its metabolites belong to the chemical family of benzimidazoles (BZM) that act as potent tubulin polymerization inhibitors, suggesting a potential re-direction of BZMs for cancer therapy. Applying UV-Vis spectrometry we here demonstrate ABZ as a DNA intercalator. This insight led us to determine the primary mode of ABZ action in mammalian cells. As revealed by RNA sequencing, ABZ did neither grossly affect replication as analyzed by survival and replication stress signaling, nor the transcriptome. Actually, unbiased transcriptome analysis revealed a marked cell cycle signature in ABZ exposed cells. Indeed, short-term exposure to ABZ arrested mammalian cells in G2/M cell cycle stages associated with frequent gains and losses of chromatin. Cellular analyses revealed ABZ as a potent mammalian spindle poison for normal and malignant cells, explaining the serious chromosome segregation defects. Since chromosomal aberrations promote both cancer development and cell death, we determined if besides its general cytotoxicity, ABZ could predispose to tumor development. As measured by loss of heterozygosity (LOH) *in vitro* and *in vivo* ABZ was found as a potent inducer of LOH and accelerator of chromosomal missegregation.

## Introduction

In 1982 the United States. Food and Drug Administration (FDA) approved Albendazole (ABZ) for the treatment of helminthiasis in humans (Dayan, 2003). Since then, ABZ developed as an affordable drug that is currently manufactured by 385 companies and distributed under at least 626 brand names listed in the Medindia’s database (Medindia, 2017). These large numbers reflect the necessity of affordable drugs in treating soil transmitted helminth (STH) as well as lymphatic filariasis infections. Pullan *et al.* provided a global estimation of STH infections for the year 2010, where around 439 million people were infected with hookworm, 819 million with *Ascaris lumbricoides* and 465 million with *Trichuris trichiura* (Pullan et al., 2014). According to the World Health Organization (WHO), approximately 947 million people in 54 countries worldwide are threatened by lymphatic filariasis (World Health Organization, 2020a) and approximately 1.5 billion people are infected with STH worldwide (World Health Organization, 2020b). Other WHO data report preventive chemotherapy of (pre)school-aged children with ABZ as a monotherapy or in combination with another drug, in many areas of the world. Altogether these data highlight the global challenge of STH to mankind, the current importance of ABZ chemotherapy in fighting STH infections in humans, and its extensive global use (Supplementary Figure S1) (for more information we like to refer readers of this study to the WHO website (World Health Organization, 2020c).

The pharmacokinetics, dynamics, and mode of action of ABZ are well described (Dayan, 2003). ABZ is a low aqueous solubility drug and therefore poorly absorbed from the gastrointestinal tract, with an absorption rate in humans less than 5% (Rigter et al., 2004). Nevertheless, administration with enhancers like fatty meals and grapefruit has been reported to increase its solubility and bioavailability (Rigter et al., 2004). ABZ and its metabolites belong to the chemical family of benzimidazoles (BZM) that act as potent tubulin polymerization inhibitors (Ramírez et al., 2007). BZM based compounds inhibit microtubule dynamics, an essential process in the formation of a functional spindle apparatus and in the proper distribution of the sister chromatids during mitosis. This marked cytotoxic feature classifies BZM as effective spindle poisons (Ramírez et al., 2007). These activities suggest a potential re-direction of BZMs for cancer therapy. We and others previously reported on the potential use of ABZ as an anti-cancer drug (Pourgholami et al., 2010; Hanušová et al., 2015; Castro et al., 2016). While spindle poisons are effective in cancer treatment, ABZs action on the mammalian spindle apparatus is poorly understood (Manchado et al., 2012). Therefore, we here followed an unbiased approach to characterize the mode of action of ABZ in transformed as well as non-transformed mammalian systems.

To ensure that during mitosis the duplicated genome is equally distributed over the daughter cells, several checkpoints are operative. Defects in the spindle apparatus predispose to checkpoint activation, chromosome segregation errors, and apoptosis. These processes form the molecular basis of cancer therapy with spindle poisons. Physiologically, these checkpoints critically contribute to genome maintenance. Inhibition of mitotic proteins like polo-like kinase 1 (PLK1), Aurora kinase A (AURKA) and Aurora kinase B (AURKB) leads to chromosome segregation errors, aneuploidy, polyploidy and micronuclei formation (Lens et al., 2010). Under conditions of spindle poisoning or checkpoint inhibition, whole chromosomes can easily be gained or lost. In healthy individuals, these gain and losses usually provide a selective growth disadvantage to the daughter cells, explaining why oncogenic site effects have not been found experimentally in ABZ treated rats nor in ABZ treated patients (Dayan, 2003; Sheltzer et al., 2017). In contrast, patients suffering from heterozygous inactivating mutations of tumor suppressor genes, such as Familial Adenomatous Polyposis-FAP (APC), Seckel Syndrome (ATR), Blooms Syndrome (BLM), Familial Breast or Ovarian Cancer Syndrome (BRCA1/BRCA2), Lynch Syndrome (MLH1/MSH2), Neurofibromatosis Type 1 (NF1), Familial Retinoblastoma (RB), and Li-Fraumeni Syndrome (TRP53) are expected to be at particular risk. Loss of the corresponding wild type allele by loss of the entire or part of a chromosome can trigger tumor development. As such, spindle poisons are expected to accelerate loss of heterozygosity (LOH) of tumor suppressors and hence cancer development, particularly in this predisposed patient group.

A PubMed search on genotoxic studies of ABZ in mammalian cells is limited to a few reports (Mantovani, 1992; Oztas et al., 2007; Tweats et al., 2016). While these reports suggest an overall genotoxicity of ABZ, conclusions drawn are quite divergent. For example, ABZ gave clear negative results in the Ames test (for review see (Dayan, 2003)) whereas other tests noted an activity of ABZ in inducing micronuclei, which often arise as a consequence of chromosomal aberrations and missegregation (Ramírez et al., 2007). Of note, ABZ was independently found to display strong teratogenicity in rats and sheep (Dayan, 2003). Clearly, as pointed out by Dayan in this retrospective evaluation of ‘old’ drugs (Dayan, 2003), the present experimental non-clinical data about activity, toxicity, and kinetics would be considered inadequate based on strict application of today’s professional and regulatory guidelines. Because ABZ is a relatively old, widely used drug with a potential anti-cancer activity, we decided to further analyze its drug safety by providing a systematic analysis on its potential genotoxicity. We here demonstrate ABZ as a potent inducer of chromosomal missegregation, the molecular basis of aneuploidy, a hallmark of cancer.

## Materials and Methods

### Ethics-Statement

All experiments were performed in accordance to Dutch and European guidelines. Protocols were approved by the local Animal Ethical Committee (IVD Instantie voor dierenwelzijn) at The Netherlands Cancer Institute, Amsterdam, The Netherlands, and the CCD (Centrale Commissie Dierproeven, the national central commission for animal experimentation) located in Den Haag, The Netherlands under 9.2.8175, where 9 is the CCD number, 2 the CCD subgroup number, and 8175 the IVD number.

### Mice

Mice were housed at room temperature and a relative humidity of approximately 55% in disposable individually ventilated cages (dIVC, Innovive®). Mice were fed Transbreed (E) PL MIN (Special Diet Services) pellet nutrition and water (Aquavive®) ad libitum. *Msh2* ^*+/−*^ and *Msh2* ^*+/+*^ (WT) male and female mice on the FVB background were identified by genotyping using allele specific PCR primers on DNA extracted from toe biopsies P1: 5′-CGG​CCT​TGA​GCT​AAG​TCT​ATT​ATA​AGG-3′, P2: 5′-GGT​GGG​ATT​AGA​TAA​TGC​CTG​CTC​T-3′, P3: 5′-CCA​AGA​TGA​CTG​GTC​GTA​CAT​AAG-3′ (De Wind et al., 1995).

ABZ powder was brought in suspension 0.5% Sodium Carboxy-methylcellulose + 1% Tween-80. Temozolomide (TMZ) was prepared as described previously (Wojciechowicz et al., 2014). Mice were exposed either to ABZ or vehicle alone by oral gavage in the morning followed by a subsequent oral treatment with TMZ in the afternoon on indicated days.

### Immunohistochemistry

Two weeks after the last ABZ exposure, mice were sacrificed using CO_2_ and intestines were fixed in 4% formaldehyde and embedded in paraffin for tissue sectioning. After deparaffinization, tissue sections were treated with TRIS/EDTA pH 9.0 to allow antigen retrieval. Endogenous peroxidases were inactivated using 3% H_2_O_2_ in methanol. Slides were blocked with PBS containing 4% bovine serum albumin (BSA) and 5% normal goat serum (NGS). Slides were incubated overnight with a MSH2-specific mouse monoclonal IgG antibody (Calbiochem; NA27) in PBS containing 1% BSA and 1.25% NGS. After washing, slides were incubated with a rabbit-anti-mouse IgG1, IgG2a and IgG3 monoclonal antibody (Abcam; ab133469). MSH2 specific immune-complexes were visualized by Labeled Polymer-HRP Anti-Rabbit Envision (DakoCytomation; K4011) using DAB (Sigma; D-5905), and rinsed with demi water. Sections were then counterstained with haematoxylin, washed in tap water and mounted with Entallan (Sigma; 1.07960). All washing in between steps was performed using PBS containing 0.05% Tween 20, unless otherwise indicated. Slides were scanned on the ScanScope^®^ XT slide scanner (Aperio, Leica Biosystems) and analyzed using Aperio ImageScope software (Aperio, Leica Biosystems). The absolute number of MSH2 deficient crypts was counted along the entire length of the small intestine.

### Interactions with Calf Thymic-DNA

CT-DNA binding was evaluated by UV-Vis spectrometry. Absorption scanning was done using CT-DNA (40 µM) and ABZ (10 µM). Spectra were obtained by reading the absorption from 230 to 320 nm (TECAN Infinity M200). Variations of absorption as well as the displacement of the wavelength of spectral maximum absorption were evaluated (Kubota et al., 1999).

CT-DNA intercalation was evaluated by fluorescence measurements at excitation/emission wavelengths of 492 nm and 620 nm. Compounds able to intercalate into DNA compete with ethidium bromide causing fluorescence reduction. CT-DNA (10 µM) was saturated with ethidium bromide (20 µM) in 50 mM phosphate buffer containing NaCl (0.1 M), pH 7.4 and ABZ was added at 10, 20, 30 and 40 µM (Da Silveira et al., 2011).

### Survival

DNA damage tolerant (DDT) and DNA damage intolerant (DDinT) cells were cultured in RPMI medium supplemented with 8% of Fetal Bovine Serum (FBS), 100 U/ml Penicillin, 100 μg/ml Streptomycin, 50 µM 2-Mercaptoethanol, 200 μM L-arginine (Buoninfante et al., 2018). HeLa, U2OS and RPE-1 cell lines were cultured in DMEM supplemented with 8% FBS, 100 U/ml Penicillin, 100 μg/ml Streptomycin, and 2 mM L-glutamine. All cell lines were incubated at 37°C and 5% CO_2_. Cells were exposed to increasing concentrations of ABZ and after 24 h cells were harvested and resuspended in PBS/1% BSA/0.02 µM Sodium Azide buffer containing 1 μg/ml propidium iodide (PI) and measured using FACSArray (Becton Dickinson). The data were analyzed with FlowJo™ vX 0.7 software following the gating strategy as described (Wit et al., 2015). The complete procedure was repeated to generate three biological replicates. Comparison between the groups was performed by Analysis of Variance (ANOVA) comparing the groups with the control.

### RNA-Seq

Sample preparation: DDT lymphoma cells were plated (10^7^ cells) in Petri dishes and treated with ABZ (400 nM), EtBr (400 nM) or mock treated for 12 h. Hereafter, the cells were lysed in Trizol and frozen for subsequent processing. The complete procedure was repeated to generate three biological replicates for each treatment. Quality and quantity of the total RNA was assessed by the 2,100 Bioanalyzer using a Nano chip (Agilent). Only RNA samples having an RNA Integrity Number (RIN) > 8 were subjected to library generation.

Library preparation: Strand-specific cDNA libraries were generated using the TruSeq Stranded mRNA sample preparation kit (Illumina) according to the manufacturer’s protocol. The libraries were analyzed for size and quantity of cDNAs on a 2,100 Bioanalyzer using a DNA 7500 chip (Agilent), diluted and pooled in multiplex sequencing pools. The libraries were sequenced as 65 base single reads on a HiSeq2500 (Illumina).

Pre-processing: Strand-specific RNA reads (11–33 million reads per sample), 65 bp single-end, were aligned against the mouse reference genome (Ensembl build 38) using Tophat (version 2.1, bowtie version 1.1). Tophat was supplied with a Gene Transfer File (GTF, Ensembl version 77) and was supplied with the following parameters: “--prefilter-multihits–no-coverage-search–bowtie1–library-type fr-firststrand.” In order to count the number of reads per gene, a custom script which is based on the same ideas as HTSeq-count has been used. A list of the total number of uniquely mapped reads for each gene that is present in the provided Gene Transfer Format (GTF) file was generated.

Analysis: For checking the data quality and performing statistical analysis, we used different packages including limma, edgeR and GoSeq (Robinson et al., 2009; Young et al., 2010). All the analyses were performed in R language (version 3.5.1). Only relevant samples were used for the differential gene expression analysis using edgeR package under default arguments with the design set to either of the two conditions form control, ABZ and EtBr treatments. Genes were considered to be differentially expressed when the False discovery rate (FDR) was below 0.001 after the Benjamini-Hochberg multiple testing correction. Sets of differentially expressed genes in indicated conditions were called ‘gene signatures. MA plots were generated after differential gene expression analysis carried by edgeR package (Robinson et al., 2009; McCarthy et al., 2012).

### Cell Cycle

DDT lymphoma cells were exposed to different concentrations of ABZ treated for 24 h, fixed in 70% ethanol, and stored at −20°C. On day of analysis, cells were re-suspended in PBS and treated with RNAse-A (100 μg/ml) for 20 min and subsequently re-suspended in PBS containing 5 μg/ml PI and measured using FACScalibur (Becton Dickinson). The data were analyzed using the FlowJo™ vX 0.7 software (Wit et al., 2015).

### Cell Imaging to Detect Microtubule Dynamics

HeLa (7.5 × 10^4^/well) cells were plated in 48 well-plates and after adherence were treated with ABZ (400, 800 and 1,600 nM), Paclitaxel (1 µM), Nocodazole (825 nM) or Noscapine (25 µM) for 12 h. The cells were fixed for 20 min (formaldehyde 4%, Triton X 0.5% in PBS). Cells were incubated overnight at 4°C with antibodies against *α*-tubulin (Sigma, 1:10,000) and crest (Cortex Biochem, 1:5,000) followed by 2 h of incubation with secondary antibody (Molecular probes, 1:500) and DAPI at room temperature. All antibodies were diluted in PBS/Tween 0.2%. The images were acquired using a Deltavision deconvolution microscope (Applied Precision) with a 60x (NA 1.42) oil objective (Olympus). Softworx (Applied Precision), ImageJ, Adobe Photoshop and Illustrator CS6 were used to process acquired images.

### Loss of Heterozygosity *in Vitro*

LOH analyses were performed using mouse embryonic stem cells (mESC) with a heterozygous deletion of the MLH1 allele (MLH1^wt/−^) and cultured as previously described (te Riele, 2009; Aarts and te Riele, 2010). The 6-thioguanine (6-TG, 400 nM) treatment is very toxic for mismatch repair (MMR) proficient- but not MMR-deficient cells, providing a simple measure for LOH (De Wind et al., 1995). For each condition 10^6^ mESC cells were plated in 10 cm Petri dishes. After 24 h, cells were exposed to 100 nM and 200 nM ABZ or 200 nM reversine, a potent inhibitor of Monopolar Spindle 1 (MPS1) for 24 h. Mock treated cells were used as negative control. After 3 days of treatment with ABZ or Reversine, the 6-TG (400 nM) treatment was started. New medium containing fresh stock of 6-TG was refreshed after every 4^th^ day. Colony formation was photographed after 15 days.

## Results

### ABZ Interacts with DNA and Competes with EtBr

**FIGURE 1:**
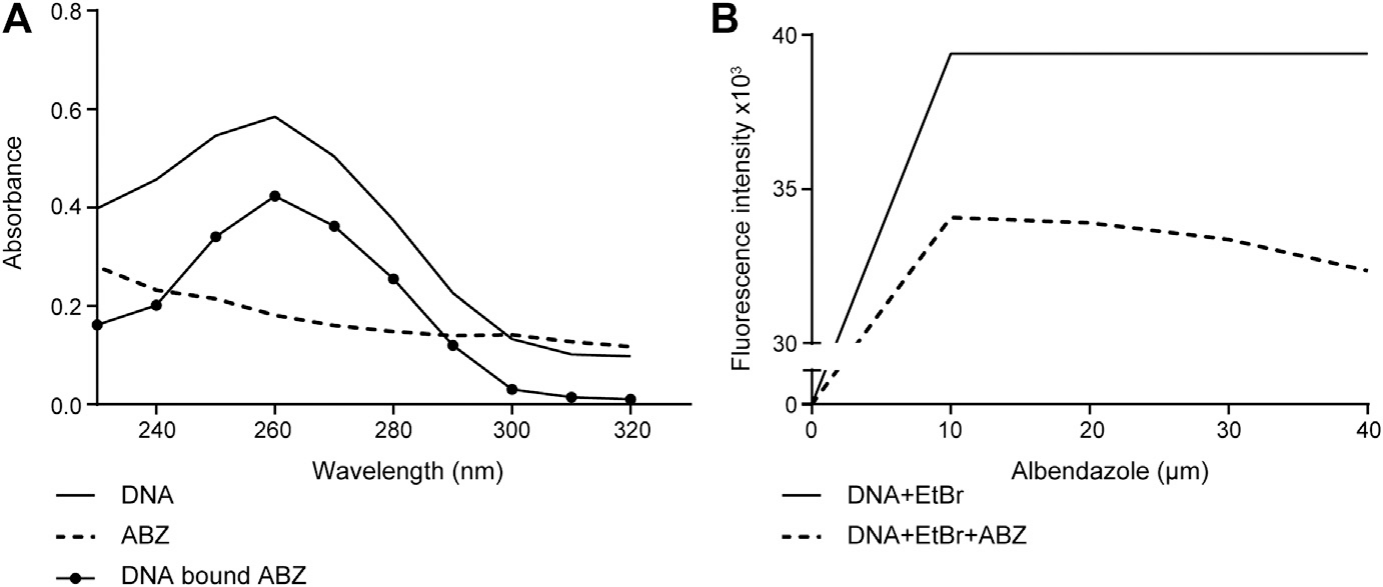
Albendazole interacts with DNA and competes with the DNA-intercalator EtBr. **(A)** Evaluation of ABZ (10 μM) binding to purified DNA (40 μM) by UV-Vis spectrometry (reading the absorption from 230 to 320 nm). **(B)** The fluorescence intensity of CT-DNA (10 μM) stained with EtBr (20 μM) is reduced by ABZ (10, 20, 30 and 40 μM). Fluorescence intensity was measured at excitation/emission wavelengths of 492 nm and 620 nm (n = 2).

The pharmacological features of ABZ have been widely studied. While ABZ acts as a tubulin polymerization inhibitor in helminth, its effect in mammalian cells is poorly understood. Given the small planar molecular structure of ABZ we considered that ABZ might actually intercalate into DNA (Kubota et al., 1999; Kubota et al., 2002). To test this possibility, we first used hypochromism as a read out for ABZ DNA binding properties and its potential intercalating activity. Hypochromism is generally consistent with the forces of interaction by intercalation, as this connection involves stacking interactions between an aromatic chromophore and DNA base pairs. As shown in Figure 1A, ABZ reduced the absorbance of purified DNA at defined wavelength. This hypochromic effect suggested that ABZ may in fact intercalate with DNA. To verify intercalation as potential type of DNA interaction we took advantage of the fact that intercalating DNA compounds usually induce hypochromism and bathochromic displacement (Shahabadi and Moghadam, 2012). If ABZ is a DNA intercalator, one predicts that ABZ competes with the DNA intercalator ethidium bromide (EtBr), a widely applied fluorescent dye to visualize and measure DNA. Indeed, ABZ was found to compete efficiently with EtBr and thereby decreased EtBr specific fluorescence (Figure 1B). This bathochromic displacement effect further indicated ABZ as a DNA intercalator, a finding in accordance with its small planar molecular structure.

### ABZ Does not Induce Replication Stress

**FIGURE 2:**
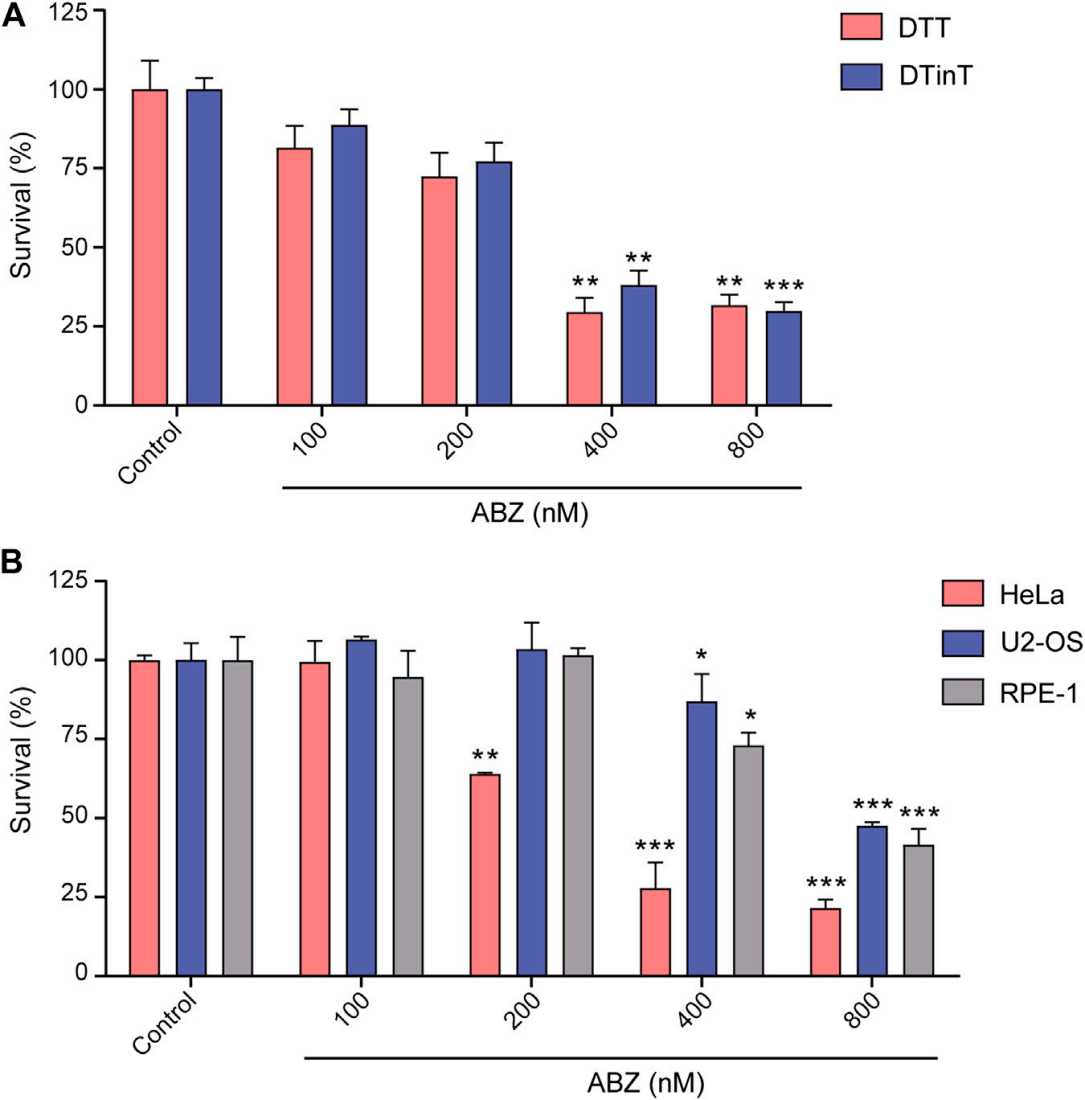
Albendazole affects cell survival but does not induce replication stress. **(A)** Evaluation of DDT and DDinT lymphoma cell lines survival upon treatment with different concentrations of ABZ. **(B)** Survival of HeLa, U2-OS and RPE-1 cell lines upon exposure to different concentration of ABZ. Data represent the mean of three independent experiments. Values are expressed as mean ± SEM (n = 3). *, ** and *** denote statistical difference of ABZ treated cells compared to non-treated cells of the control when *p* < 0.1, *p* < 0.01 and *p* < 0.001, respectively. Comparison between these groups was performed by Analysis of Variance (ANOVA).

To determine if the non-covalent interaction of ABZ with DNA mimics DNA damage and blocks replication, we took advantage of a unique isogenic set of wild type, i.e. DNA damage tolerant (DDT) and PcnaK164R mutant, i.e. DNA damage intolerant (DDinT) thymic lymphoma cell lines established from a p53^ko^;*Pcna* ^*K164R/loxP*^ mutant mouse (Langerak et al., 2007; Buoninfante et al., 2018). DDT and DDinT lymphoma cells were exposed to increasing concentrations of ABZ. Both cell lines were found to be equal sensitive to ABZ, suggesting that ABZ does not grossly interfere with DNA replication, at least not in a manner that sensitizes DDinT cells (Figure 2A). We conclude that ABZ does not interfere at the level of replication. Most likely, as intercalators interact non-covalently with DNA, they are efficiently removed during DNA unwinding.

To extend our studies to the human system, we determined the toxicity of ABZ on human transformed cell lines as well as non-transformed immortalized cell lines. Similar to murine lymphoma cells, both cell lines were found hypersensitive to ABZ. The slight differential sensitivity threshold of transformed HeLa cells (400 nM) as compared to immortalized RPE-1 cells and transformed U2-OS (800 nM) likely relates to differences in the rate of proliferation (Figure 2B). Apparently, the toxicity of ABZ on murine and human cells is quite similar.

### Transcriptome of ABZ Exposed Cells

**FIGURE 3:**
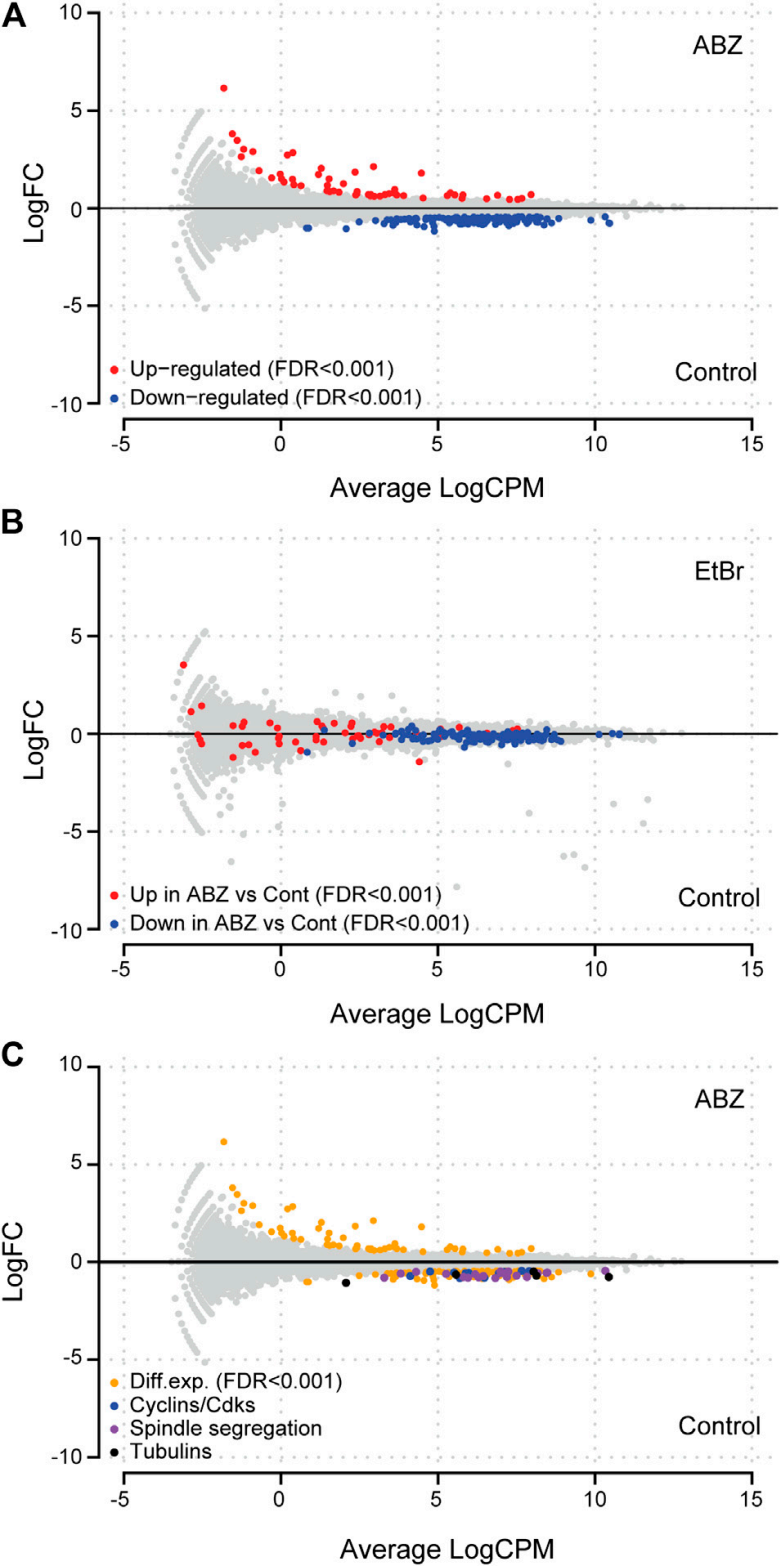
Albendazole affects genes linked to mitosis. **(A)** MA-plot of lymphoma cell lines exposed to ABZ or a vehicle control. Differentially up- or down-regulated genes (FDR < 0.001) are shown in red and blue, respectively. **(B)** ABZ and EtBr differentially affect the transcriptome. MA-plot of lymphoma cell lines exposed to EtBr or a vehicle control showed that the genes up- or down-regulated by ABZ, are not affected by EtBr. **(C)** Exposure to ABZ affects transcription of cyclins, cyclin dependent kinases, DNA damage response, spindle, segregation, and tubulin genes (for a list of shown genes in each subgroup, see Supplementary Table S2).

As ABZ and EtBr intercalate with DNA, we questioned whether and in which way EtBr and ABZ affected gene expression patterns. To approach this question in a genome-wide and an unbiased manner, we exposed the previously mentioned DDT lymphoma cell line to ABZ (400 nM), EtBr (400 nM), or to DMSO, as a vehicle control. After exposure, cells were harvested, total RNA was isolated and used for RNA-Seq analysis. Subsequently, specific or generic changes in the transcriptome pertaining to ABZ or EtBr alone were defined by comparing ABZ and EtBr dataset to the DMSO control. First, we determined the ABZ signature by defining specific transcripts that are either induced or suppressed by ABZ (Figure 3A, red and blue labeled respectively, and Supplementary Table S1. Remarkably, the majority of transcripts that were differentially expressed in the ABZ setting remained unaffected by EtBr, except for three genes: Idi1, Ccne2 and Aacs, which were found downregulated in both conditions. Likewise, the EtBr signature appeared entirely different from ABZ (Figure 3B). A closer analysis of the ABZ signatures (Figure 3C) revealed a marked decrease of genes directly linked to mitosis. This observation suggested that critical mitotic genes are expressed prior to chromosome condensation and the overrepresentation of mitotic arrested cells in the ABZ condition result in an underrepresentation of these transcripts.

### ABZ Affects Cell Cycle Progression and Causes Chromosomal Missegregation

**FIGURE 4:**
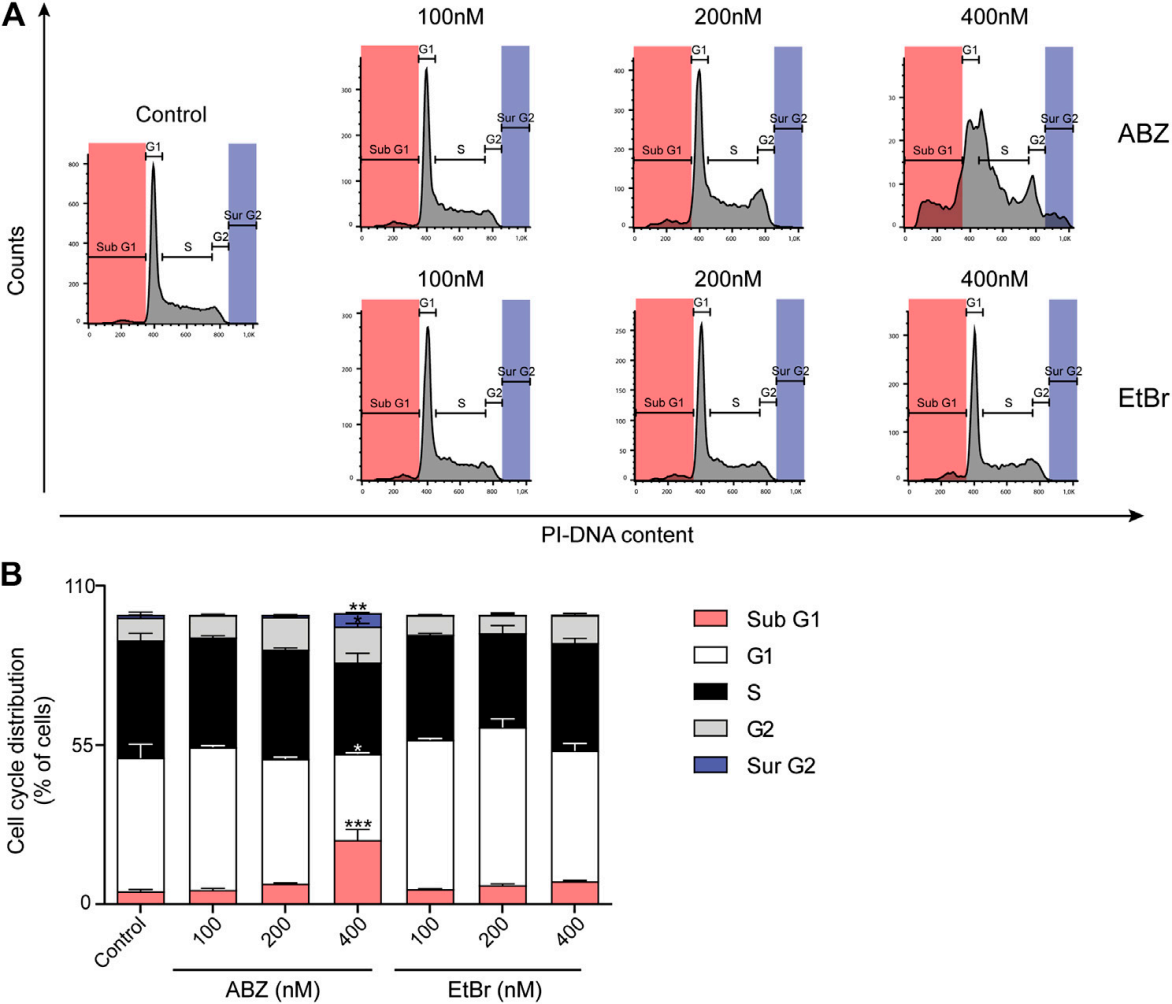
Albendazole affects cell cycle progression and causes chromosomal aneuploidy. **(A)** Flow cytometric analysis of cell cycle progression in the presence of ABZ or EtBr (concentrations are indicated). Data are representative of two experiments with two independent cell lines. **(B)** The relative cell cycle distribution of DDT lymphoma cells in the presence of ABZ or EtBr. The averages of DDT cell line are shown (Mean ± SD).

The pronounced underrepresentation of mitotic genes in the ABZ exposed samples led us to analyze the impact of ABZ on the cell cycle. Increasing doses of ABZ resulted in a gradual increase of cells arrested in the G2/M phase. This effect was associated with a dose dependent increase of viable aneuploid cells (Figure 4). Apart from subG1 cells, this aneuploidy was characterized by the frequent occurrence of surG2 cells, pointing to a critical role of ABZ in promoting chromosomal segregation errors. This phenomenon was not observed for EtBr.

### ABZ Poisons the Mammalian Spindle Apparatus

**FIGURE 5:**
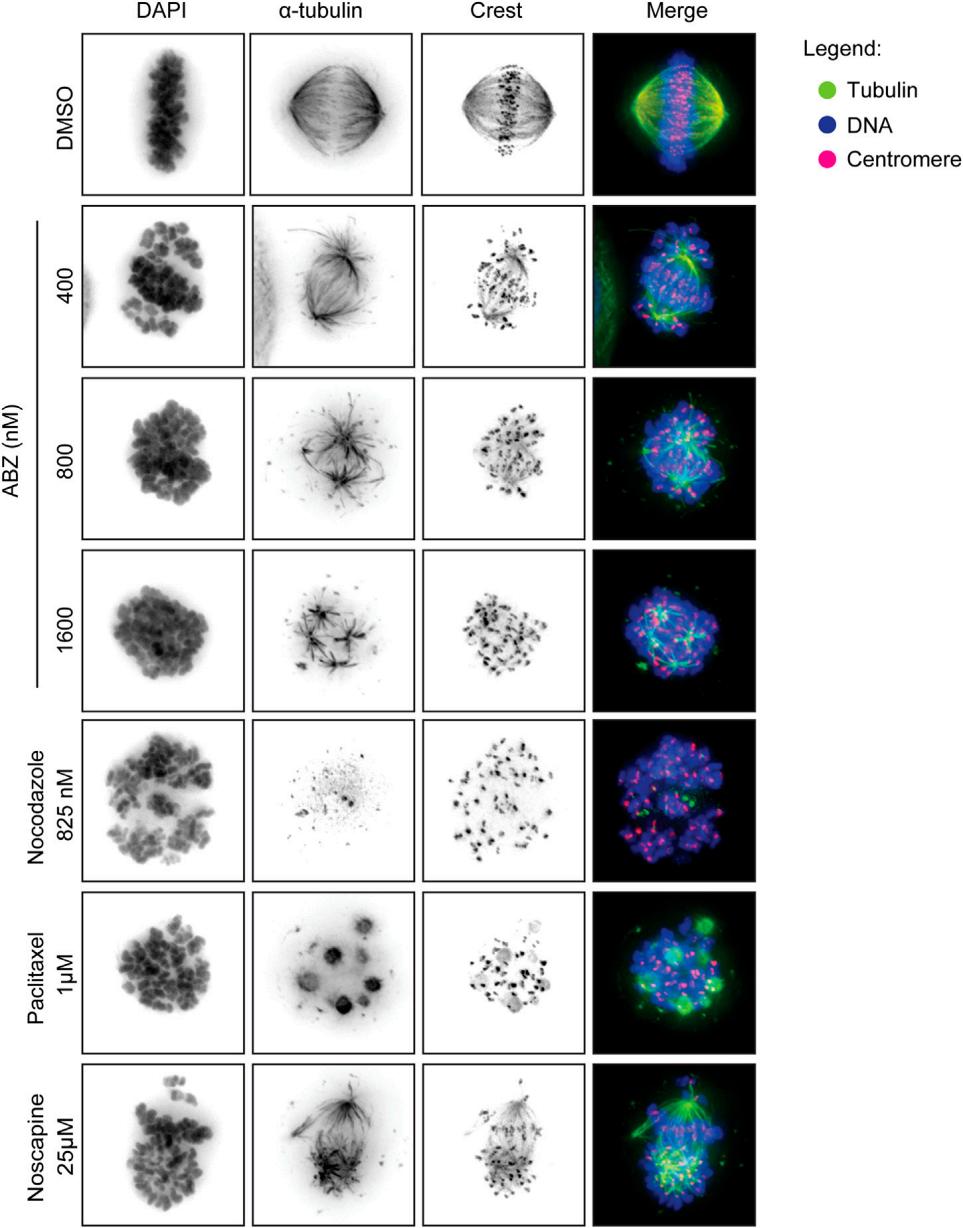
Albendazole induces spindle apparatus disruption. ABZ act as spindle poison for mammalian cells, disrupting the spindle apparatus. HeLa cells were exposed to the indicated drugs, fixed and stained with an anti-alpha tubulin antibody (green) and an anti-centromere antibody (crest, red). DAPI was used to stain the DNA. The images were acquired by wide-field fluorescence microscopy.

To measure any direct effect of ABZ on the human spindle apparatus, we exposed HeLa cells to ABZ. As positive controls for spindle poisons we selected Paclitaxel as a typical microtubule stabilizer, Nocodazole as a microtubule polymerization inhibitor, and Noscapine as a spindle poison that affects microtubule dynamics (Figure 5). After 12 h cells were fixed and the spindle apparatus was revealed with α-tubulin specific antibodies (green), and centromeres with CREST specific antibodies (pink). DNA was revealed by DAPI staining (blue). As visualized by wide-field fluorescence microscopy (Figure 5), ABZ was found as a potent mammalian spindle poison, inducing misalignments. Mitotic figures most closely resembled those of Noscapine, with the marked notion that the ABZ effect was achieved at a molar concentration that was 60-fold lower as compared to Noscapine. These observations explain the high frequency of aneuploidy in cells treated with low doses of ABZ.

Taken together, ABZ acts as a strong spindle poison and potent inducer of aneuploidy, both in helminth as well as mammals, thereby inhibiting cell cycle progression in both systems. Transcriptional alterations induced by ABZ appeared largely indirect.

### ABZ Strongly Stimulates Loss of Heterozygosity *in vitro*

**FIGURE 6:**
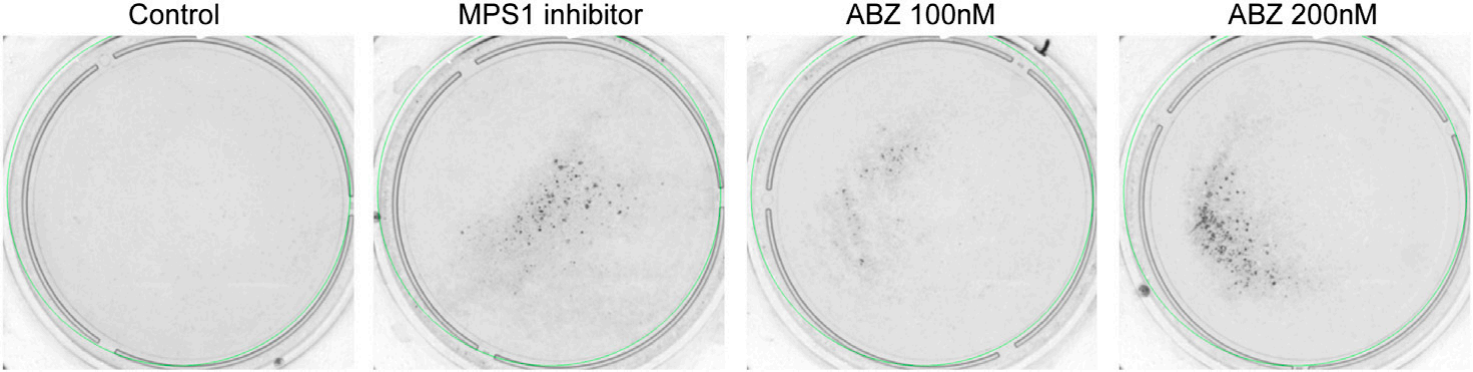
Albendazole stimulates loss of heterozygosity *in vitro*. mES cells haploinsufficient for the mismatch repair (MMR) component *Mlh1* (*Mlh1* ^*wt/−*^) were plated (10^6^ cells/dish) in the presence and absence of ABZ. After 24 h, ABZ was removed. The cells subsequently were exposed to 6-TG. Cells that lost MMR activity because of LOH events form 6-TG-resistant colonies. Reversine (200 nM, a potent inhibitor of MPS1) was used as positive control.

The sum of these data implicated a high risk for ABZ in inducing chromosomal missegregation. As chromosomal missegregation is a hallmark of cancer, we questioned if ABZ could promote aneuploidies in general and especially in tumor-prone patients that display a haploinsufficiency for a specific tumor suppressor gene. If this is the case, one expects that ABZ exposure stimulates loss of heterozygosity (LOH). To address this critical issue, we took advantage of an *in vitro* approach enabling a direct measure of the LOH frequency (De Wind et al., 1995). Embryonic stem (ES) cells haploinsufficient for the mismatch repair gene *Mlh1* were cultured in the presence and absence of ABZ for 24 h. Three days later, LOH cells were selected by exposure to 6-thioguanine (6-TG) over a time course of fifteen days. 6-TG specifically kills mismatch repair (MMR) proficient cells while MMR deficient cells can resist low doses of 6-TG. Hereafter, surviving mismatch repair deficient colonies, i.e. those that lost the remaining functional *Mlh1* allele, were determined for different concentrations of ABZ. Remarkably, a low concentration of ABZ sufficed to induce LOH (Figure 6).

### ABZ Is a Potent Stimulator of LOH *in vivo*

**FIGURE 7:**
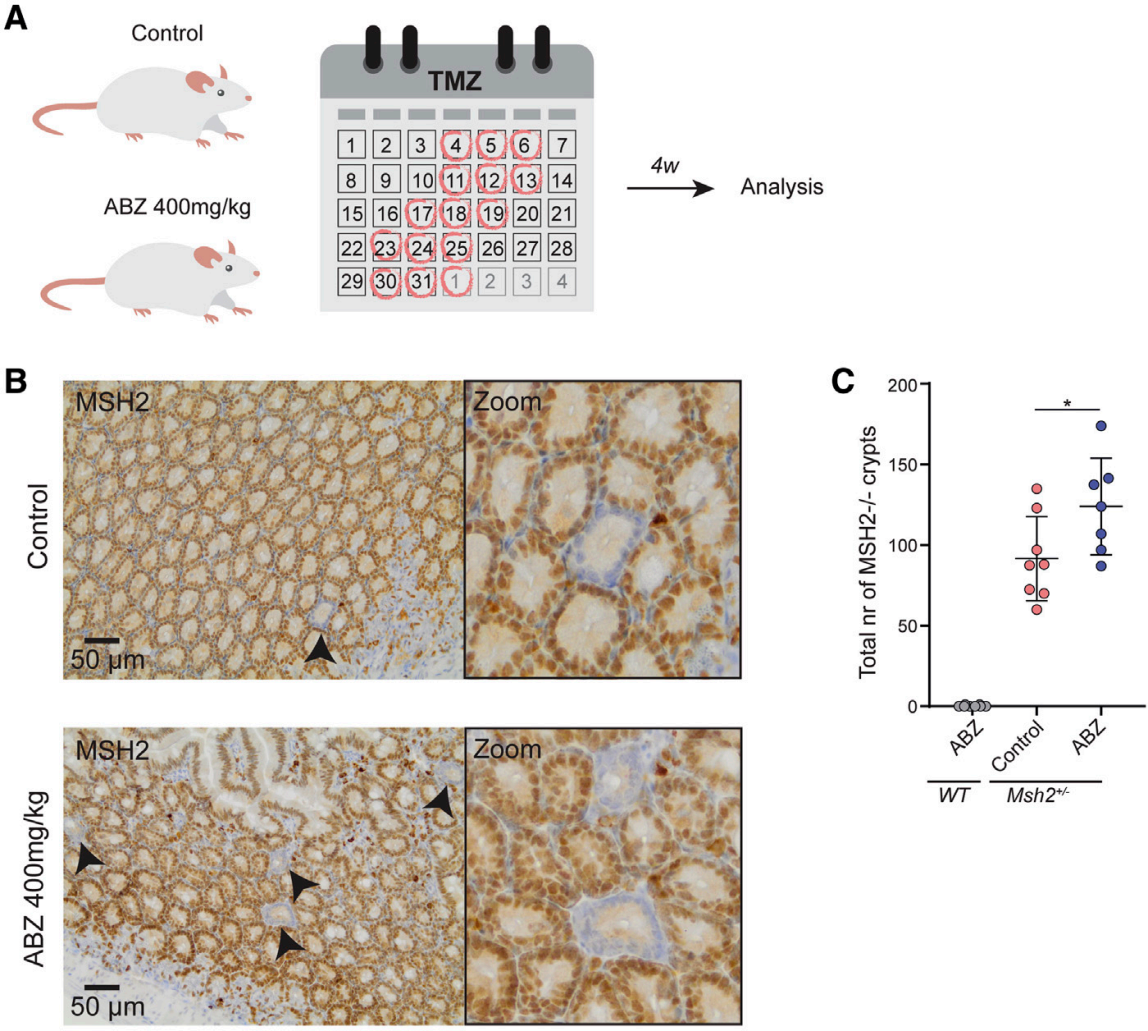
Albendazole accelerates LOH in intestinal stem cells *in vivo*. **(A)** *In vivo* experimental set up. *Msh2* ^*+/−*^ mice were exposed to ABZ (400 mg/kg) (n = 7) or control solution (n = 8) by oral gavage for 32 consecutive days. Every 3–4 days this was followed by subsequent administration of TMZ (100 mg/kg) by oral gavage for three consecutive days. After a two-week rest period, mice were sacrificed and intestines were fixed. **(B)** Immunohistochemical staining of small intestine for MSH2. **(C)** Quantification of **B**. Individual MSH2-deficient crypts along the entire length of the small intestine were counted manually. WT mice exposed to ABZ were used as negative control (n = 8). Plotted are the mean and SD, asterisk indicates *p* value <0.05 (One way ANOVA).

The fact that ABZ stimulates LOH *in vitro*, led us to address whether ABZ could also induce the same effect *in vivo*. To accomplish this goal, we took advantage of *Msh2* heterozygous (*Msh2* ^*+/−*^) mice, a model for Lynch Syndrome (LS), in which LOH could be addressed *in vivo*. In LS patients, MMR deficient crypt foci are commonly found in the intestinal tract, which have the potential to progress into tumors (De Wind et al., 1995; Kloor et al., 2012). As chromosome mis-segregation generally generates a cell cycle arrest and chromosome gains or losses of larger chromosomes commonly function as tumor suppressors (Duijf et al., 2013; Santaguida et al., 2017; Sheltzer et al., 2017), and in particular a loss of MSH2 does not confer a direct proliferative advantage to the cells, we provided MSH2-deficient cells a selective advantage by exposure to the methylating agent Temozolomide (TMZ). In short, mice were exposed daily to ABZ or carrier solution only, for 32 consecutive days, while every week this treatment was followed by subsequent TMZ administrations for three consecutive days (Figure 7A). Hereafter, we scored MSH2-deficient crypts from immunohistochemical staining covering the entire small intestine (Figure 7B). Quantification of the number of MSH2 deficient crypts along the entire length of the small intestinal tract showed a significant increase in the ABZ treated group, indicating that ABZ stimulates LOH *in vivo* (Figure 7C).

## Discussion

Since the FDA approval of ABZ in 1982 for the treatment of helminthiasis in humans, ABZ developed as an affordable drug that is currently manufactured and distributed by 385 companies, reflecting the necessity of affordable drugs in treating STH as well as lymphatic filariasis infections (Dayan, 2003). Although teratogenicity of ABZ has been reported in sheep and rats (Dayan, 2003), the precise molecular impact of ABZ on mammalian, and in particular humans remains unclear. Given this uncertainty, pregnant woman are advised not to use ABZ. However, as patients may be unaware of a pregnancy, a potential teratogenic risk of ABZ usage might occur. Strikingly, recent reports suggest a re-direction of ABZ in cancer treatment (Hanušová et al., 2015; Castro et al., 2016; Ghasemi et al., 2017; Varbanov et al., 2017; Priotti et al., 2018). These new insights prompted us to investigate the molecular impact of ABZ on mammalian cells. Despite the fact that ABZ interacts with DNA and competes with EtBr, ABZ appeared to have no direct impact on replication and transcription. Yet, the remarkable sensitivity of human and murine transformed and non-transformed cells to ABZ led us to study potential alterations in the transcriptome of ABZ exposed cells.

Similar to EtBr, the effects of ABZ on the transcriptome appeared indirect. While our results and those of others (Piechota et al., 2006; Lee et al., 2008) revealed that EtBr poisons mitochondrial genes, the transcriptional profile of ABZ pointed to a marked increase of cells arrested in G2/M. These observations led us to determine the effect of ABZ on cell cycle progression. In line with the ABZ specific transcriptome profile, cells exposed to ABZ arrested in a dose dependent manner at G2/M, which is consistent with previous findings in various cancer cell lines (Ghasemi et al., 2017; Zhang et al., 2017). Furthermore, the ABZ induced cell cycle arrest was associated with a marked dose dependent increase in the frequency of viable cells with chromosomal aneuploidy, which is likely caused by the potent inhibitory activity of ABZ on tubulin polymerization, an essential process during mitosis (Dawson et al., 1984; Chu et al., 2009). These results question the preferential targeting of helminth tubulin by ABZ (Lacey, 1988; Hanušová et al., 2015). In fact, our study indicates that ABZ toxicity in helminths and mammalian cells follows the same molecular principle, as has been reported by others (Chu et al., 2009; Ghasemi et al., 2017; Zhang et al., 2017).

To determine the impact of ABZ on tubulin polymerization, de-polymerization and spindle dynamics, we here set out to determine the toxicity of ABZ on the mammalian spindle apparatus in independent human cell lines. ABZ acted as a strong spindle poison, which resulted in the accumulation of cells in the G2/M phase in the cell cycle, explaining not only the typical ABZ transcriptome signature but also the effective and rapid induction of aneuploidy *in vitro*. While tissue specific patterns of aneuploidy can prohibit or initiate cancer development (Albertson et al., 2003; Fröhling and Döhner, 2008; Stratton et al., 2009; Sack et al., 2018), our observations led us to perform LOH studies in the presence of ABZ. Again, consistent with the high frequency of aneuploid cells upon ABZ exposure, ABZ was found to be remarkably efficient in inducing LOH in mouse embryonic stem cells. Only 100 nM of ABZ sufficed to effectively induce LOH. Interestingly, these observations made *in vitro* also applied to *in vivo* settings, where ABZ accelerated the formation of MSH2 deficient intestinal crypts in *Msh2* heterozygous mice. Though we appreciate that the dose of ABZ used in our *in vivo* experiments is higher than the dose that is given to humans, the poor solubility of ABZ might give rise to high local concentrations in the gut. Nevertheless, future studies should address whether our observations also apply to conditions where lower doses of ABZ are continuously administered. The fact that the tumor incidence did not rise abruptly in the general population of human ABZ users, supports the concept that a chromosomal aneuploidy is detrimental to a cell, and not oncogenic (Sheltzer et al., 2017). However, our data suggests this may be different in the context of tumor suppressor gene haploinsufficiency. To examine the putative tumor promoting properties of ABZ in a predisposed genetic background, future studies may take advantage of the *Apc* ^*+/*min^ mouse model (Yamada and Mori, 2007).

Our results argue against prophylactic anti-helminth treatment of haploinsufficient carriers of tumor suppressor genes. These include patients suffering from Familial Adenomatous Polyposis-FAP (APC), Seckel Syndrome (ATR), Blooms Syndrome (BLM), Familial Breast or Ovarian Cancer Syndrome (BRCA1/BRCA2), Lynch Syndrome (MLH1/MSH2), Neurofibromatosis Type 1 (NF1), Familial Retinoblastoma (RB), and Li-Fraumeni Syndrome (TRP53). The observations made in this study are likely to apply to other tubulin poisons used to treat helminthiasis, such as Ivermectin (IVM), Praziquantel (PZQ), and Metronidazole (MNZ) (Fennell et al., 2008). Of note, these drugs have recently been reconfirmed and categorized by the WHO as important, most essential medicines in the treatment of helminthiasis and specific bacteria.

The sum of these insights indicates that ABZ should be prescribed with caution to haploinsufficient carriers of tumor suppressor genes. Furthermore, it highlights the need for developing more selective drugs in treating helminthiasis.

## Data Availability

The RNA-Seq reported in this article have been deposited at the National Center for Biotechnology Information under the accession number GSE163419 (https://www.ncbi.nlm.nih.gov/geo/query/acc.cgi?acc=GSE163419).
