# Supplementary material for: The Widely Used Antihelmintic Drug Albendazole is a Potent Inducer of Loss of Heterozygosity
Source: Front Pharmacol. 2021 Feb 18;12:596535. doi: 10.3389/fphar.2021.596535 (PMC7935534; doi:10.3389/fphar.2021.596535)

Supporting Figure S1

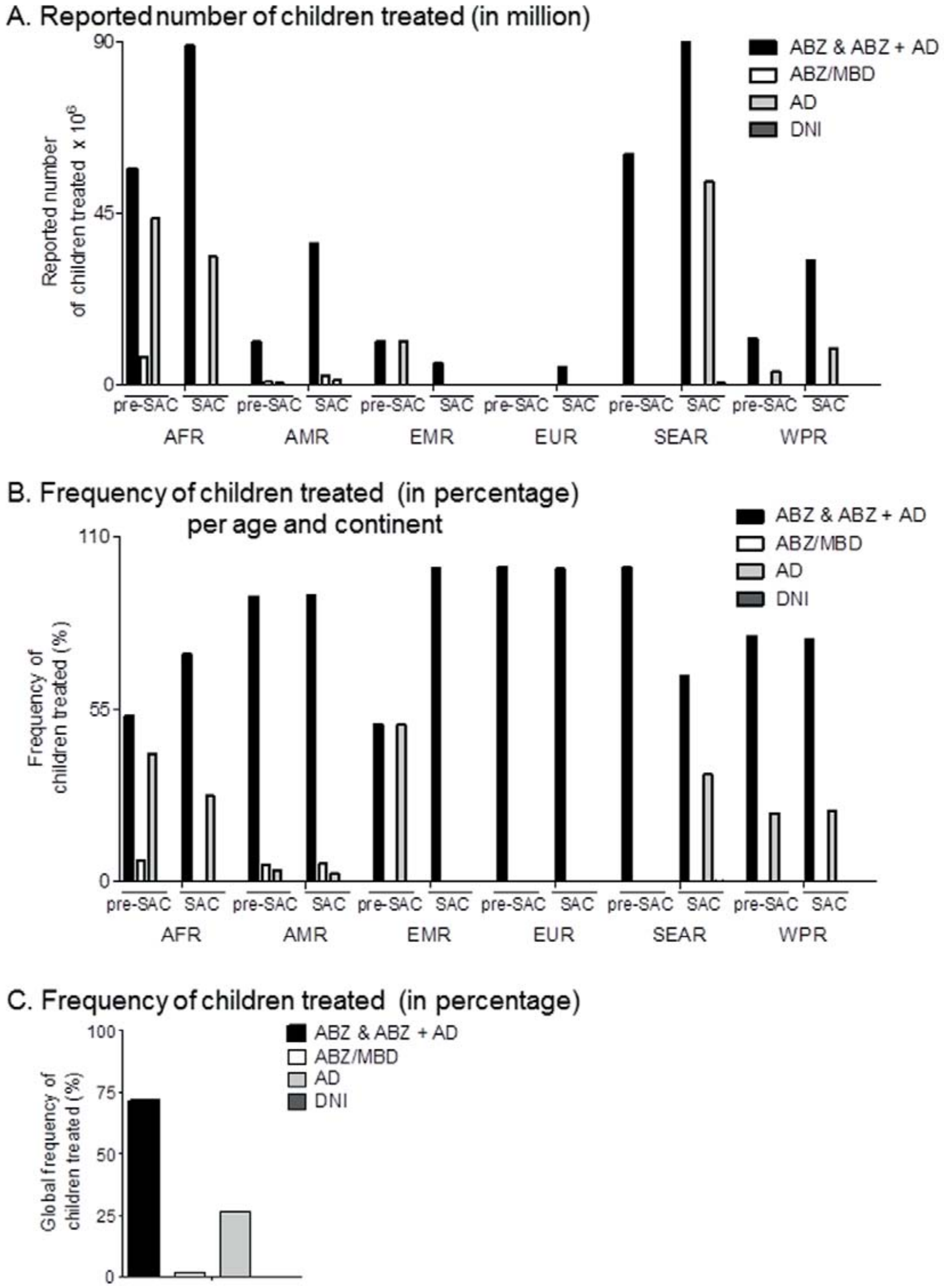

Supplement: Supplementary file 3 [file image1.pdf]
