# Supplementary material for: The Widely Used Antihelmintic Drug Albendazole is a Potent Inducer of Loss of Heterozygosity
Source: Front Pharmacol. 2021 Feb 18;12:596535. doi: 10.3389/fphar.2021.596535 (PMC7935534; doi:10.3389/fphar.2021.596535)
Supplement: Supplementary file 4 [file datasheet1.docx]

**SUPPLEMENTARY DATA**

**S1 Fig. World distribution of helminthiasis treatment for children during 2015** (based on WHO Soil-transmitted helminthiasis: Country x indicators). (A). Reported number in millions of children treated with ABZ or another drug. (B). Frequency of children treated with ABZ or another drug sorted by age and continent. (C) Frequency of reported number of children treated with ABZ or another drug. ABZ: Albendazole; ABZ + AD: ABZ in combination with another drug; ABZ/MBD: ABZ or Mebendazole; AD: another drug; DNI: drug not informed; Pre-SAC: Pre-School Age Children; SAC: School Age Children; AFR: Africa; AMR: Americas; EMR: Eastern Mediterranean; EUR: Europe; SEAR: South-East Asia; WPR: Western Pacific.

**Table S1. Differentially expressed genes in DDT lymphoma cells upon Albendazole exposure as compared to mock treatment**

**Table S2. List of mitosis related differentially expressed genes in Albendazole vs mock treated DDT lymphoma cells**
